# Pan-Genomic Identification and Analysis of the Maize BBX Family

**DOI:** 10.3390/genes17010046

**Published:** 2025-12-31

**Authors:** Xingyun Wang, Senan Cheng, Baijuan Du

**Affiliations:** CIMMYT—China Shandong Maize and Wheat Research Center, College of Agronomy, Shandong Agricultural University, Tai’an 271018, China; m15610419338@163.com (X.W.); chengsenan@163.com (S.C.)

**Keywords:** BBX family, cold stress, maize pan-genome, transcriptome data analysis

## Abstract

BBX transcription factors play crucial roles in plant growth, development, and stress resistance. Utilizing maize whole-genome data, we identified 35 members of the maize *BBX* gene family, comprising 18 core genes, 14 near-core genes, 4 non-essential genes, and 150 private genes. The phylogenetic tree constructed using Arabidopsis thaliana revealed that the fourth subfamily contained the largest number of core genes, totaling eight, and exhibited significant diversity throughout the evolutionary process of maize. The Ka/Ks ratios of the BBX family members in the 26 genomes indicated that, except for *ZmBBX20* and *ZmBBX42* under positive selection, the remaining genes were subjected to purifying selection. Further analysis combining transcriptome data and RT-qPCR demonstrated that maize BBX family member expression levels changed significantly in response to cold stress after cold treatment, highlighting their important roles in abiotic stress responses. In summary, in this study, we utilized the maize pan-genome and bioinformatics approaches to investigate maize BBX family member evolutionary relationships and functional roles, providing a new theoretical framework for further research on this gene family.

## 1. Method

### 1.1. Identification of BBXs in the Maize Pan-Genome

The genomic sequences and annotation data for the 26 maize genomes were obtained from Hufford et al.’s study [1], and the Hidden Markov Model [2] configuration file for the B-box domain (PF00643) was retrieved from the Pfam database [3]. The BBX genes were identified using HMMER3.3.2 [2], with an e-value threshold of e < 1 × 10^−5^. Candidate BBX genes were submitted to InterPro (https://www.ebi.ac.uk/interpro/about/interproscan/ accessed on 24 July 2025), and proteins without a BBX domain (PF00643) were subsequently removed, allowing for definitive identification of the maize BBX gene family members.

### 1.2. Phylogenetic Analysis of and Presence/Absence Variation in ZmBBX Gene Family

To classify maize BBX proteins, we constructed a phylogenetic tree using BBX proteins from Arabidopsis and maize, with the former sequences obtained from the [4] database. Multiple sequence alignments were conducted using Prank (v.170427) [5], the phylogenetic tree was constructed with IQTREE v2.1.4 [6] with the following parameters: (iqtree2 -s At_BBX_domain_aligned.fasta -m MFP -B 1000 --bnni -T 10 --prefix At_BBX.domain), and the tree was visualized using iTOL [7]. The presence/absence variation (PAV) data for BBX genes were obtained from a previous study [8]. The PAV dataset can be downloaded from the following link: https://download.maizegdb.org/Pan-genes/Pan-Zea/pan-zea.v2.pan-genes_table.tsv.gz.

### 1.3. Expression Profile Analysis of Maize BBX Genes

The public database PPRD [9] (http://ipf.sustech.edu.cn/pub/zmrna/) was used to download the expression data (FPKM) of the B73 inbred in different tissues and under cold conditions, and a heatmap was created in R software [10]. Specific expression datasets were obtained from PRJNA171684 (SRX172727-SRX739036) [11] and PRJNA344653 (SRX2195945-SRX2195957, SRX2195984-SRX2195996, SRX2196024-SRX2196029) [12]. The heatmap is generated using the Pheatmap R package 4.2.2, and the expression data is used after being log2 transformed.

### 1.4. Gene Ontology (GO) Enrichment Analysis

The clusterProfiler package (v4.10.0) in R software v4.3.2 was used for Gene Ontology (GO) enrichment analysis [13]. GO terms with a corrected *p*-value < 0.05 were considered to be significantly enriched. The gene co-expression network was visualized using Cytoscape v3.8.0 (http://cytoscape.org/) [14].

### 1.5. Plant Material and Stress Treatment

Maize seeds were sown in pots (30 × 20 × 14 cm) filled with a nutrient-soil-to-vermiculite mixture at a 2:1 (*v*/*v*) ratio. The trays were supplemented daily with 2 L of water, and the plants were cultivated in a greenhouse maintained at 28 °C with 60% relative humidity and a light intensity of 2000 lx for 14 days. For the cold treatment, maize plants were initially grown under normal conditions for 14 days, followed by exposure to 4 °C in a Lihen growth chamber under a photoperiod of 16 h light/8 h darkness for 2–4 days. After cold treatment, the seedlings were allowed to recover at 25 °C for 2 d [15].

### 1.6. RT-qPCR

Total RNA was extracted from the leaves of 14-day-old seedlings using TRIzol reagent (Invitrogen, Carlsbad, CA, USA). Subsequently, RNA was reverse-transcribed into cDNA using a reverse-transcription kit. Quantitative PCR analysis was performed using SYBR Green reagent (Takara, Kusatsu, Japan) on a Roche LightCycler system, while quantitative real-time PCR was performed on an ABI StepOnePlus real-time PCR system (Applied Biosystems, Foster City, CA, USA) using SYBR Green reagent (Takara Bio, Inc., Kusatsu, Japan) to detect specific genes. Relative expression levels of the target genes were calculated using the 2^−ΔΔCt^ method [16], with the maize 18S gene serving as an internal reference for data normalization.

## 2. Introduction

Plant growth and development are highly complex and precisely coordinated biological processes regulated by internal genetic programs and external environmental signals. Transcription factors are key molecular regulators that precisely control the expression of downstream genes at the transcriptional level by specifically binding to cis-acting elements in target genes’ promoter regions, thereby mediating various physiological and biochemical responses. Among these, the B-box (BBX) protein family represents an important class of zinc finger transcription factors, characterized by the presence of one or two typical B-box domains at the N-terminus [17]. Some members also contain a CCT (CO, CO-LIKE, and TOC1) domain at their C-termini [18]. These characteristic domains collectively determine the protein–protein interaction capabilities and biological functions of BBX proteins.

B-box proteins are a class of zinc finger transcription factors characterized by zinc finger domains composed of histidine, cysteine (Cys), and zinc ions [17,19]. These domains form multiple finger-like projections that can bind to metals such as zinc, and proteins with them typically contain specialized interaction domains that enable them to bind to DNA and perform their functions [20,21]. BBX family transcription factors contain one or two B-box domains, and some possess a CCT (CONSTANS, CO-like, and TOC1) domain. They play important roles in plant growth, development, flowering, and environmental adaptation [22,23].

BBX proteins are classified into five distinct subfamilies according to the number of B-box and CCT domains. B-box domains are classified into two types (B-box1 and B-box2) based on disparities in their amino acid sequences and zinc ion coordination specificity [24]. Despite these minor structural variations, their molecular functions are highly conserved across species. Subfamilies I and II both possess two B-box domains and one CCT domain [25], but are distinguished by the second B-box domain’s type. In contrast, Subfamily III is characterized by a simpler architecture, comprising only one B-box domain and one CCT domain; IV contains two B-box domains but no CCT domain; and V contains only one B-box domain [26]. Some BBX transcription factors also contain VP motifs [17]. Studies have demonstrated that the B-box domain is involved in protein–protein interaction [27], whereas the nuclear localization signal is contained within the CCT domain. Numerous reports have indicated that the CCT domain is associated with transcriptional activation [25,28].

Transcription factors of the BBX family are key regulators of plant growth and development. They mediate photomorphogenesis, flowering regulation, and responses to abiotic stress. Studies have shown that in Arabidopsis thaliana, BBX proteins regulate photomorphogenesis through the COP1-HY5 pathway [29,30,31,32]. The flowering process is controlled by multiple signaling pathways. BBX transcription factors mediate photoperiodic flowering regulation. In Arabidopsis, *co* loss-of-function mutants show delayed flowering, while CO overexpression causes precocious flowering [33]. However, under short-day conditions, *co* mutants show no difference in flowering time compared to the wild type, while overexpression still leads to precocious flowering. This indicates that CO dosage is a critical factor influencing flowering [34]. In tomato, *SiBBX7*, *SiBBX9*, and *SiBBX20* contribute to enhanced cold tolerance by preserving the integrity and function of photosystem II (PSII) under chilling stress [35]. In apple, *MdBBX37* directly integrates into the ICE1-CBF regulatory pathway to enhance cold tolerance [36]. Furthermore, BBX transcription factors may mediate the integration of light signaling and temperature perception, participating in the cold acclimation of Arabidopsis in response to light signals [37].

In this study, we analyzed 26 maize pan-genome datasets and identified 48 *BBX* gene family members, which were categorized into core, near-core, and dispensable genes according to their prevalence across germplasms. Transcriptomic profiling under cold stress revealed pronounced expression reprogramming in multiple *BBX* genes, implicating their role in cold tolerance via modulating specific transcriptional networks. We further pinpointed two BBX transcription factors as key candidates mediating cold stress responses, likely functioning through downstream signaling pathways dependent on their canonical B-box domains. These results offer important mechanistic insights into BBX-regulated abiotic stress responses in maize and suggest promising targets for enhancing crop stress resilience.

## 3. Result

### 3.1. Identification and Evolutionary Analysis of the ZmBBX Transcription Factor Family

**Figure 1:**
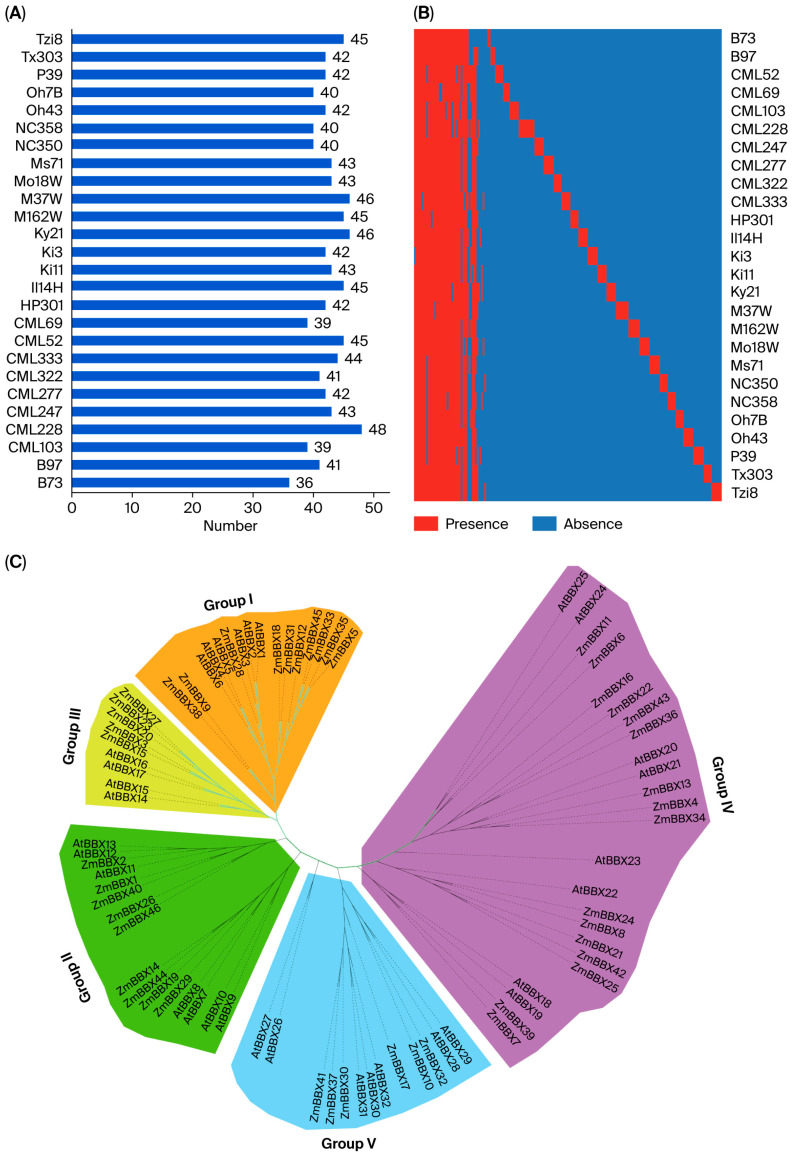
Identification and phylogenetic analysis of ZmBBXs in pan-genome. (**A**) Number of ZmBBXs. (**B**) Heatmap of presence and absence of 35 ZmBBXs in 26 maize varieties, except for core genes. (**C**) Phylogenetic tree of BBXs from Arabidopsis and maize.

Based on the Arabidopsis BBX family unified nomenclature provided by Khanna et al. [20], 35 ZmBBX family members have been identified in the pan-genome of maize. Using B73 as the reference genome, BBX family member numbers and protein sequence lengths were compared across different maize inbred lines to reveal their variations. In terms of BBX family member numbers, 36 ZmBBXs were identified in both CML52 and MS71, whereas 48 were identified in CML228 (Figure 1A). Based on their distribution across different inbred lines, BBX transcription factors can be classified into core genes, near-core genes, non-essential genes, and private genes [38]. Presence–absence variation analysis of the ZmBBX family members showed that it included 18 core genes, 13 near-core genes, and 4 non-essential genes, with 150 private genes detected (Figure 1B).

Further analysis of the different BBX family member protein domains was conducted. A phylogenetic tree was constructed based on maize and Arabidopsis domain sequences, and the family was classified into five subfamilies according to Gangappa et al. [17]. Subfamily III only included 5 core genes, the smallest number, whereas Subfamily IV contained the largest number, with 16 BBX family members, all of which were core genes. Subfamilies I, II, and V contained 10, 9, and 6 BBX family members, respectively (Figure 1C).

### 3.2. ZmBBX Is Subjected to Varying Selective Pressures in Different Maize Varieties

**Figure 2:**
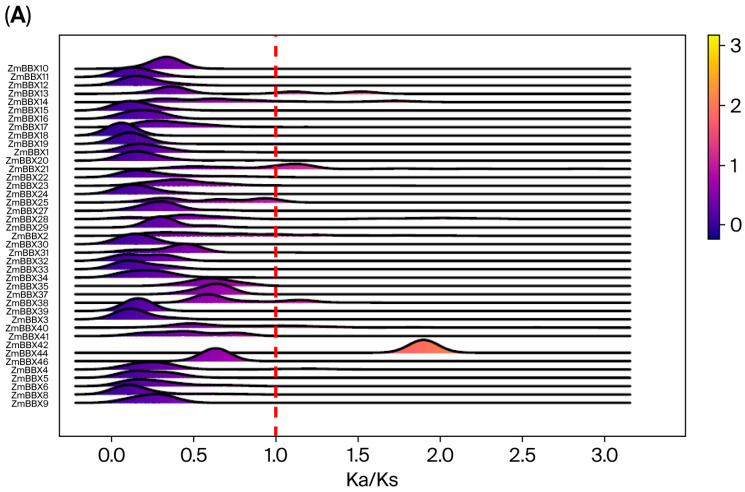

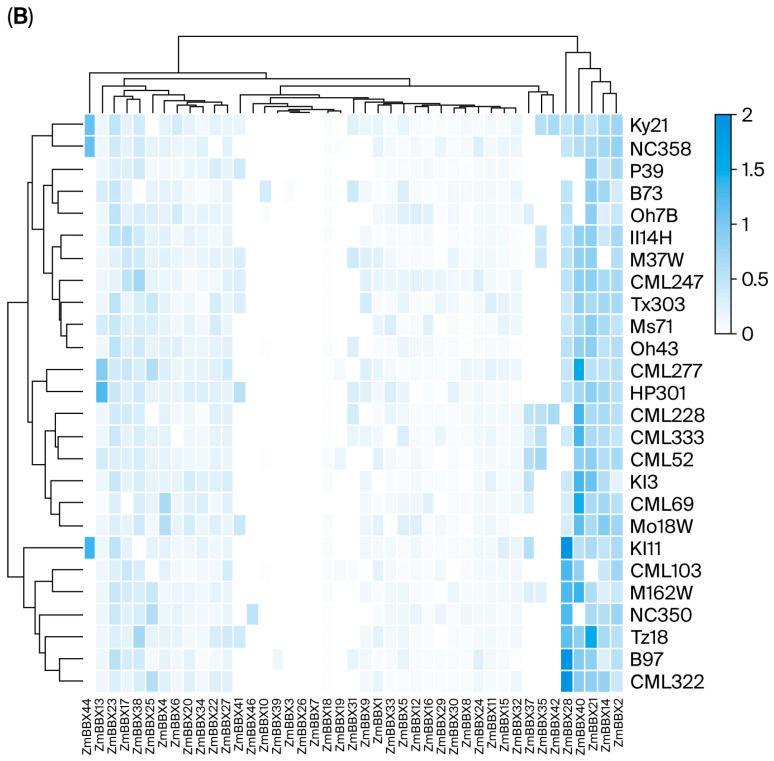
Ka/Ks values of ZmBBX. (**A**) Distribution of Ka/Ks values of ZmBBX in 26 maize varieties. (**B**) Heatmap of different maize variety occurrence frequencies at each BBX with Ka/Ks ratio > 1.

The Ka/Ks ratio is a crucial indicator for studying genetic evolutionary mechanisms [39]. To investigate the selective pressures on BBX family members across the 26 maize genomes, the Ka/Ks ratios for each family member were calculated. The results revealed that except for *ZmBBX20* and *ZmBBX44*, which exhibited peak values exceeding 1, most BBX family members’ Ka/Ks peak values were below 1 (Figure 2A). Some maize inbred lines showed a positive selection effect on *ZmBBX44*, whereas other genes were under purifying selection. Further analysis of the Ka/Ks ratio greater than 1 heatmap indicated that only *ZmBBX28*, *ZmBBX40*, and *ZmBBX44* displayed high Ka/Ks ratios, suggesting that these genes underwent selective pressure during maize evolution (Figure 2B).

### 3.3. Analysis of Promoter Cis-Acting Elements

**Figure 3:**
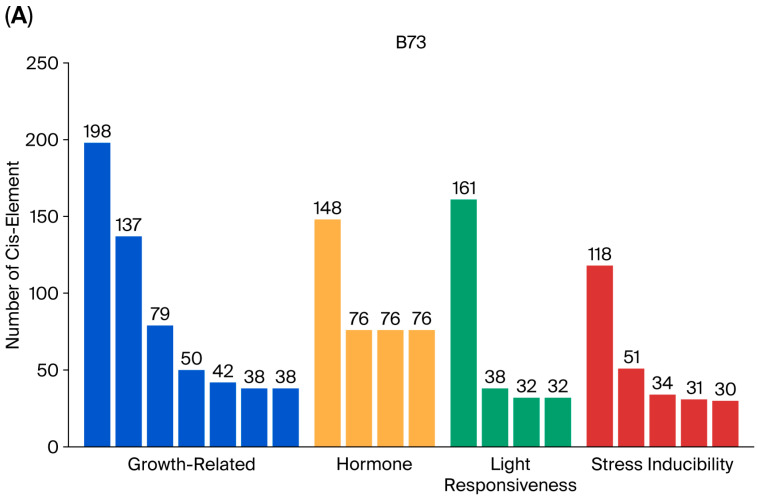

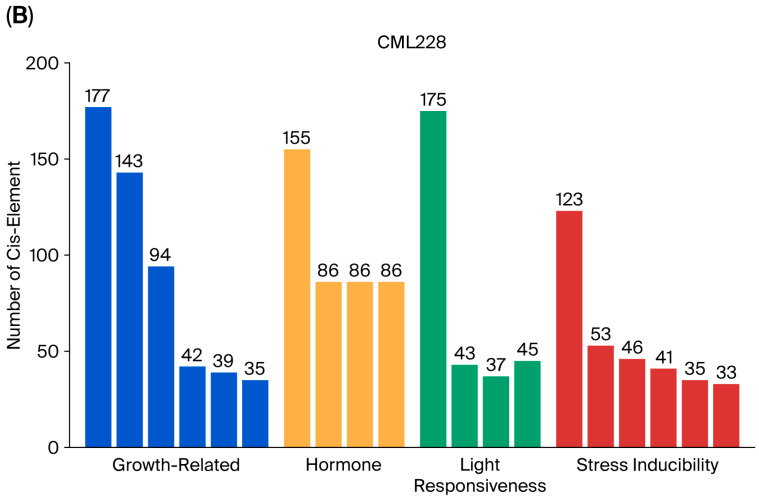
Statistics of the number of cis-acting elements in B73 and CML228. (**A**) B73 cis-acting element number statistics. (**B**) CML228 cis-acting element number statistics.

The CML228 inbred line had the highest number of BBX family members; therefore, it was chosen to subsequently analyze the promoter cis-acting elements. The top 20 genomes with the highest number of cis-acting elements were selected for statistical analysis and plotting (Figure S1), with the results showing that, in the B73 reference genome, there were seven development-related cis-elements, four hormone-related elements, four elements involved in the light response, and five stress-related elements. In contrast, the CML228 genome contained six development-related cis-elements, four hormone-related cis-elements, four cis-elements involved in the light response, and six stress-related cis-elements. These findings suggest that differences in cis-acting elements indicate that different *BBX* genes play diverse roles across inbred lines (Figure 3). For example, *BBX* genes may influence different developmental and stress resistance processes in different inbred lines.

### 3.4. Patterns of BBX Family Transcription Factors

**Figure 4:**
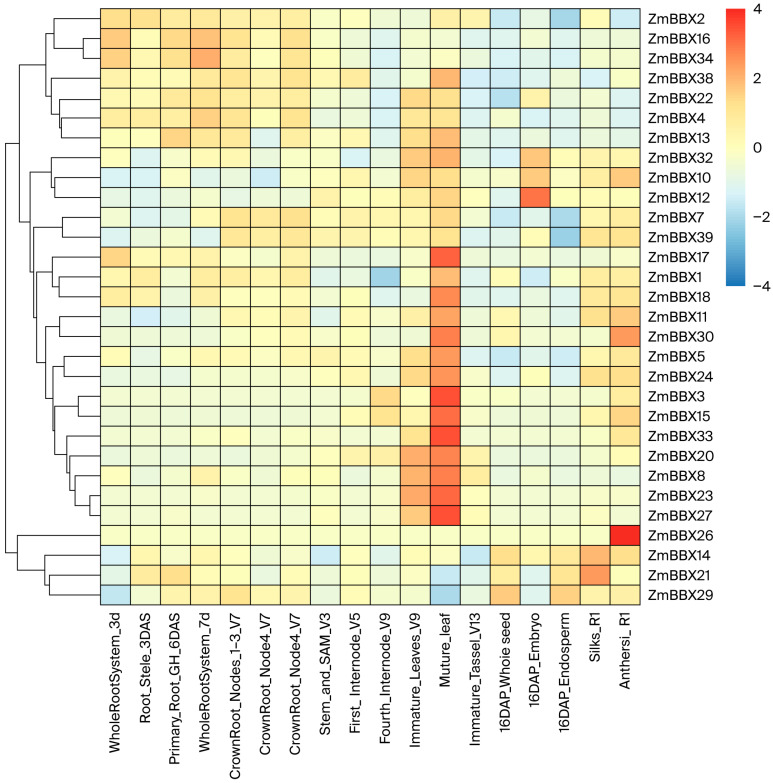
Heatmap of BBX expression associated with maize development in different tissues.

RNA-seq data from the public database PPRD were obtained before and after cold treatment to analyze BBX family gene expression patterns. The results showed that 14 BBX family members were highly expressed in mature leaves, 2 were highly expressed in anthers, and 7 were highly expressed in roots; the expression levels of other members were relatively low. Expression analysis revealed distinct tissue specificity among the BBX family members: 14 were highly expressed in mature leaves, 2 in anthers, and 7 in roots, with the remainder showing low expression. Notably, all transcription factors occupying central positions in the protein–protein interaction network were highly expressed in the leaves (Figure 4). These results indicate that BBX family transcription factors are associated with normal function in mature leaves.

### 3.5. Co-Expression Network and Enrichment Analysis of Maize BBX Family Genes

**Figure 5:**
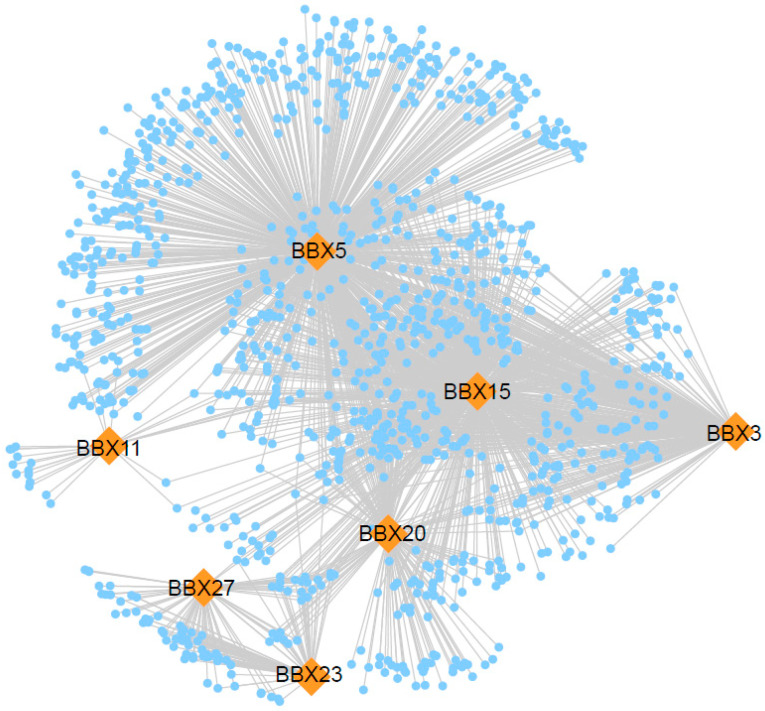
BBX family protein–protein interaction network.

BBX proteins represent a significant class of transcription factors that play crucial roles in various processes, including photomorphogenesis, flowering time regulation, biological rhythms, and responses to abiotic stress in plants. Their functions are typically mediated through forming complex regulatory networks with other factors. In this study, we employed the online platform Maize Netome, developed by Huazhong Agricultural University, to conduct a co-expression network analysis of maize BBX genes. Subsequently, we utilized Cytoscape version 3.8.0 to compute and visualize this network. Based on publicly available data analyzing co-expression correlation coefficients, we examined the co-expressed genes within the maize BBX gene family. As illustrated in Figure 5, ZmBBX5 occupies the most central position in the network, followed by *ZmBBX15, ZmBBX3*, *ZmBBX20*, *ZmBBX27*, *ZmBBX23,* and *ZmBBX11.*

**Figure 6:**
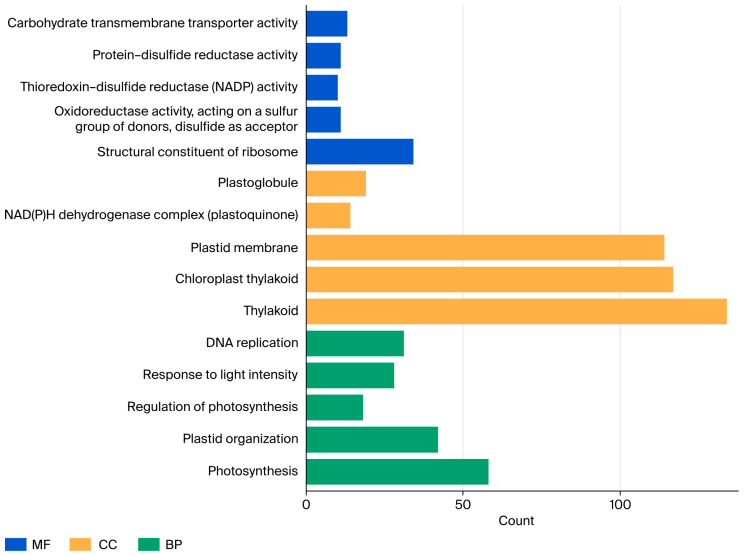
GO enrichment analysis of BBX-mediated regulatory networks in response to cold stress.

To investigate the functions of these genes, we performed Gene Ontology (GO) enrichment analysis on those within the co-expression network; the results are presented in Figure 6. In terms of cellular components, most genes are enriched in chloroplast thylakoid, plastid membrane, and NAD(P)H dehydrogenase complex. Additionally, some genes are associated with biological processes such as photosynthesis, DNA replication, and plastid organization. Regarding molecular functions, several types of oxidoreductase activity are represented, including protein-disulfide reductase activity, thioredoxin-disulfide reductase (NADP) activity, and oxidoreductase activity, which act on a sulfur group of donors. In alignment with the predominant leaf expression of most BBX transcription factors, these findings strongly imply that the BBX family plays a central role in regulating photosynthetic processes in maize (Figure 6).

### 3.6. The Role of BBX Family Transcription Factors in the Cold Stress Response

**Figure 7:**
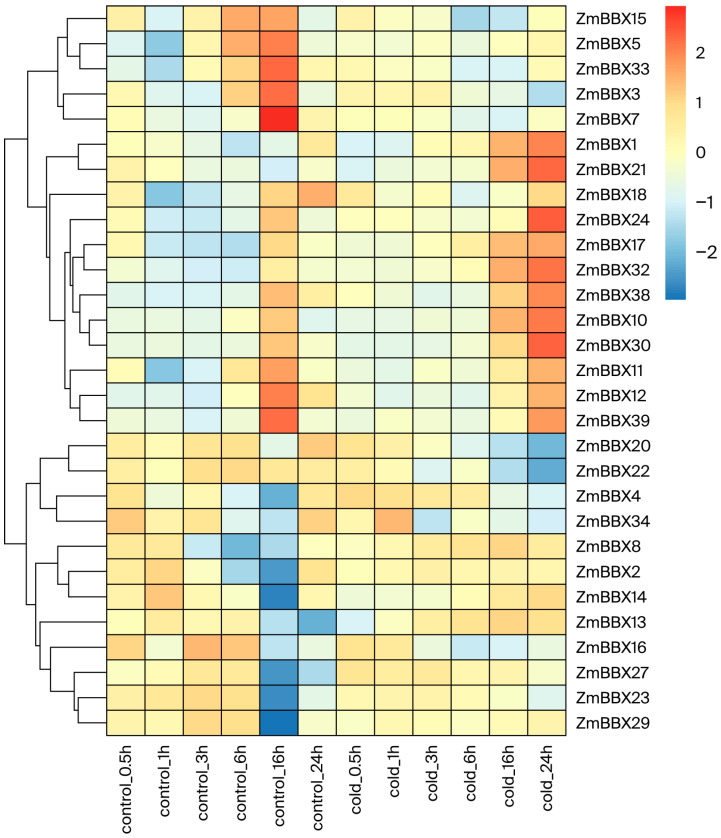
The role of BBX family transcription factors in the cold stress response.

As the primary chloroplast concentration site, maize leaves also serve as crucial organs for perceiving external environmental signals. To investigate BBX transcription factor expression patterns under cold stress, we identified BBX family members that exhibited significant changes in expression before and after 4 °C treatment. The results revealed dynamic alterations in leaf gene expression following cold exposure. Specifically, *ZmBBX3*, *ZmBBX5*, *ZmBBX7*, *ZmBBX15*, and *ZmBBX33* reached peak expression levels 26 h post-treatment before declining rapidly, suggesting their potential involvement in early responses to cold stress. Conversely, 11 genes (*ZmBBX1*, *ZmBBX21*, *ZmBBX24*, *ZmBBX17*, *ZmBBX32*, *ZmBBX38*, *ZmBBX10*, *ZmBBX30*, *ZmBBX11*, *ZmBBX12*, and *ZmBBX39*) showed elevated expression at 16 h but also maintained high expression levels at 24 h, indicating their possible roles in the later stages of cold adaptation (Figure 7). This suggests that these genes may play pivotal roles in the cold response of maize and warrant further investigation as candidate genes.

### 3.7. Response of BBX Genes to Cold Stress

**Figure 8:**
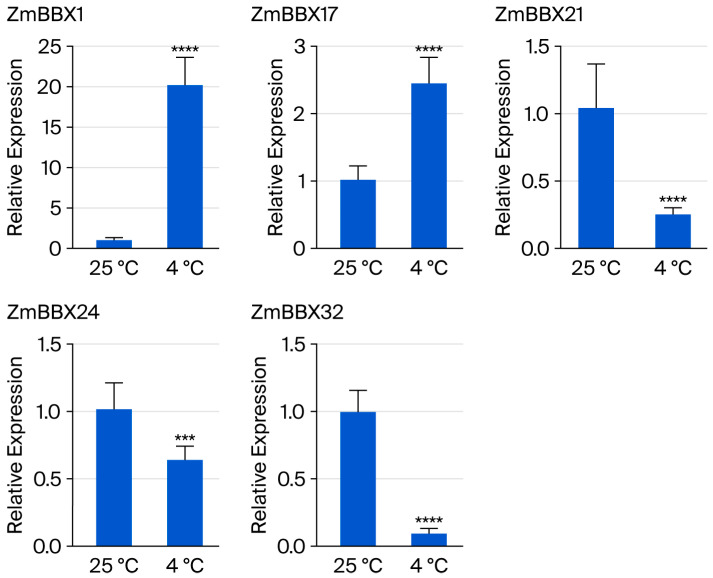
Expression profiles of five BBX genes under cold stress (the horizontal axis: time after stress treatment; the vertical axis: relative expression levels; **** *p* < 0.0001, *** *p* < 0.05, *p* > 0.0001).

Further analysis was conducted to investigate the response of *ZmBBX* genes to cold stress. The results demonstrated that, after 12 h of treatment at 4 °C, the *ZmBBX* gene expression levels showed significant differences before and after cold stress treatment (Figure 8). When maize was exposed to cold stress, *ZmBBX1* and *ZmBBX17* expression was induced and upregulated, indicating their positive regulatory roles in cold tolerance. In contrast, *ZmBBX21*, *ZmBBX24*, and *ZmBBX32* expression levels decreased under cold stress conditions, with *ZmBBX32* being the most sensitive, suggesting that *ZmBBX32* may function as a negative regulator of maize response to cold stress.

## 4. Discussion

As a typical tropical crop, maize demonstrates a cultivation range primarily concentrated in low- to mid-latitude regions. Enhancing its cold tolerance is crucial for expanding production into higher-latitude areas; therefore, low temperature represents one of the major environmental stresses limiting crop productivity. Improved cold resistance in maize would facilitate earlier spring sowing, thereby enabling more efficient utilization of early spring light and land resources.

At the molecular level, BBX transcription factors regulate target gene expression through complex formation with other regulatory proteins. Under various stress conditions, BBX proteins specifically interact with different signaling molecules and undergo conformational changes that modulate their capacity to bind to target gene promoters, ultimately activating or repressing specific genes to promote rapid plant adaptation to environmental challenges [40,41,42].

Compared to conventional single-reference-genome-based analyses, pan-genome approaches overcome reference genome limitations. Utilizing a maize pan-genome comprising 26 high-quality chromosome-level genomes, we systematically identified 196 BBX family members in this study, with only 35 core genes conserved across all accessions, indicating substantial germplasm specificity among most BBX genes.

Previous research has established BBX transcription factors as key participants in plant abiotic stress responses [43]. Under cold stress, BBX proteins enhance cold tolerance by activating antifreeze protein and other cold-responsive genes, thereby promoting the synthesis of protective compounds [44].

In our study, five BBX family transcription factors were identified as potentially involved in cold stress response in maize. GO enrichment analysis revealed that their functions are predominantly enriched in photosynthesis-related pathways. Additionally, these BBX transcription factors exhibit specific high expression in leaves, leading us to hypothesize that they may function within chloroplasts—a notion that requires further experimental validation. A similar mechanism has been reported in tomato cold tolerance, where knockout of SlBBX7, SlBBX9, and SlBBX20 significantly suppressed photosynthesis under cold stress [35].

While traditional studies have emphasized the role of BBX in direct cryoprotective mechanisms downstream of the ICE-CBF signaling pathway, our research highlights its potential function in photoprotection [36,43,45,46]. Early spring cold stress frequently coincides with high light intensity, where impaired chloroplast function leads to photoinhibition and reactive oxygen species accumulation [44,47,48]. The high leaf expression of BBX genes and their regulatory role in chloroplast development suggest that they enhance overall cold tolerance by maintaining photosynthetic apparatus integrity and stability, thereby ensuring energy supply and reducing photodamage [49,50,51]. This mechanism represents an independent yet complementary pathway to classical antifreeze responses.

Notably, this chloroplast-associated function may explain why most BBX members exhibit “non-core” gene characteristics. Maize varieties from different geographical origins likely maintain specific BBX alleles to optimize the balance between photosynthetic efficiency and cold adaptation in local environments. These findings provide both a valuable candidate gene reservoir and a theoretical framework for breeding cold-tolerant maize varieties tailored to specific ecological regions.

In summary, in this study, by leveraging maize pan-genome resources, we systematically characterize the BBX gene family’s structural features and expression patterns, elucidating its significant role in maize cold tolerance. From these findings, we establish a new theoretical foundation for understanding maize cold tolerance mechanisms and advance molecular breeding strategies.

## Data Availability

Publicly available datasets were analyzed in this study. This data can be found here: PRJNA171684 and PRJNA344653.

## Supplementary Materials

The following supporting information can be downloaded at: https://www.mdpi.com/article/10.3390/genes17010046/s1, Figure S1: Distribution of motifs on the BBX promoter.
