# Supplementary figures and images for: Pan-Genomic Identification and Analysis of the Maize BBX Family
Source: Genes (Basel). 2025 Dec 31;17(1):46. doi: 10.3390/genes17010046 (PMC12841286; doi:10.3390/genes17010046)

B73

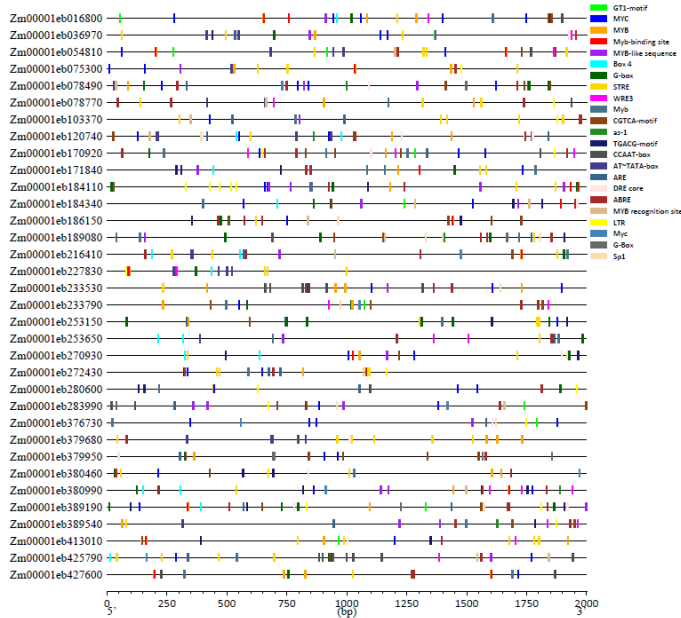

CML228

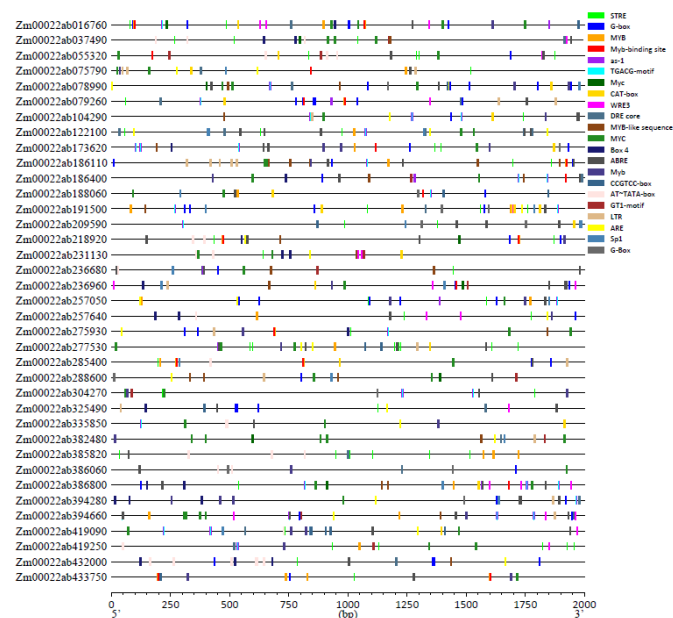

## Supplementary Figure S1

### Distribution of motifs on the BBX promoter

Supplement: Supplementary file 1 [file genes-17-00046-s001.zip › genes-4031087-supplementary.pdf]
